# Communicating COVID-19 vaccine information to Chinese communities in the UK: a qualitative study of their knowledge, information sources and trust

**DOI:** 10.1136/bmjph-2023-000658

**Published:** 2024-09-25

**Authors:** Qian Sarah Gong, Zhenghan Gao, Ian Somerville, Circle Steele, Dian Wang, Huiyu Zhou

**Affiliations:** 1 Arts, Media and Communication, University of Leicester, Leicester, UK; 2 Qingdao University of Technology, Qingdao, Shandong, China; 3 University of Leicester, Leicester, UK; 4 Wai Yin Society, Manchester, UK; 5 University of Greenwich, London, UK

**Keywords:** COVID-19, Community Health, Sociodemographic Factors, Public Health

## Abstract

**Introduction:**

In late 2020, the COVID-19 vaccine acceptance rates of Chinese people living in the UK were estimated between 52% and 57%, significantly lower than that of the general population (76%). This disparity formed a primary motivation for this study which explored Chinese communities’ overall understanding of and attitude towards the COVID-19 vaccines, the sources of information about the vaccines and levels of (dis)trust in these sources.

**Methods:**

29 focus groups with 154 participants from UK-based Chinese communities of varied sociodemographic backgrounds were conducted between March and November 2021. Focus group data were coded with NVivo and analysed using thematic analysis.

**Results:**

Participants generally had a good understanding of the health benefits of the vaccines, however, many still had concerns about vaccine safety and efficacy. They accessed COVID-19 vaccine information from a multitude of sources and had diverse information needs which to a large extent shaped their knowledge of and attitude towards the vaccines. Participants generally had good digital access and some have developed good digital literacy enabling discernment of mis/disinformation. The ways in which they accessed and engaged with various types of information sources were differentiated by diversities in country/regions of origin, years of residence and main language/dialects spoken. They also demonstrated varying degrees of trust about the communication around vaccines depending on the perceived expertise of the authority, their character and the perceived accuracy and transparency of the information.

**Conclusion:**

The UK government and health authorities need to understand Chinese communities’ diverse information needs and cultural practices to form culturally and linguistically appropriate COVID-19 vaccine communication strategies to develop trust, targeting not the entire community but subgroups within the community via credible media sources and community networks.

**WHAT IS ALREADY KNOWN ON THIS TOPIC**

- UK-based Chinese communities had higher levels of vaccine hesitancy than that of the general population. However, there is no in-depth qualitative research that explores the reasons of their vaccine hesitancy.

**WHAT THIS STUDY ADDS**

- This in-depth, qualitative study adds value and new knowledge to enquiries into ethnic minority health by researching UK-based Chinese communities and their knowledge, information sources and trust in relation to COVID-19 vaccines.

**HOW THIS STUDY MIGHT AFFECT RESEARCH, PRACTICE OR POLICY**

- This study makes a number of recommendations to UK health authorities about developing fine-tuned, culturally and linguistically appropriate COVID-19 vaccine communication strategies for ethnic minority communities.
- Understanding community members’ diverse information needs and sources, building trust and engaging with community organisations are important components of the strategies.

## Background

 The COVID-19 pandemic unleashed catastrophic damage to human populations worldwide including almost seven million reported deaths.^1^ A global effort to curb the pandemic led to the development of a number of COVID-19 vaccines by different countries including Pfizer–BioNTech (US-Germany), Moderna (US), Oxford–AstraZeneca (UK), Sinopharm and Sinovac (China) and Sputnik (Russia). With 70.5% of the world population having received at least one dose of a COVID-19 vaccine by September 2023,^2^ the successful global COVID-19 vaccination programmes have played a crucial role in containing the virus. On 5 May 2023, the WHO declared an end to COVID-19 as a global health emergency, more than 3 years after the pandemic was first declared on 11 March 2020.^3^

Despite this remarkable success, there are still a large number of people across the world who are COVID-19 vaccine hesitant.^4^,^6^ Vaccine hesitancy, defined as ‘delay in acceptance or refusal of vaccination despite availability of vaccination services’ has been identified by the WHO as a global health threat.^7^ Studies in the USA, UK and Australia suggest that vaccine hesitancy is particularly salient among ethnic minorities who already experience health disparities due to systematic and structural inequalities.^8^,^13^ Information and communication emerged as key issues because exposure to vaccine misinformation, the lack of clarity in vaccine-related information and mistrust of healthcare systems have been identified as causes of vaccine hesitancy.^14^,^16^ Ethnic minority communities are found to be disproportionately affected by COVID-19, experiencing higher rates of morbidity and mortality and greater adverse socioeconomic consequences.^17 18^ While there are some studies focusing on Black and South Asian communities,^12 14 19^ there are no in-depth studies on Chinese communities in the UK, the ethnic group which has the second highest level of vaccine hesitancy.^10^ At the end of 2020, COVID-19 vaccine acceptance rates of UK-based Chinese people were estimated between 52% and 57%, significantly lower than that of the general population (76%).^1220^,^22^ According to the latest 2021 census data, 445 646 Chinese people lived in England and Wales, constituting one of the largest non-EU ethnic groups.^23^ Given its relatively large size, the Chinese communities’ vaccine hesitancy and potential low uptake could increase the risk of virus transmission within its communities and the risk of prolonging the pandemic for the whole society. However, UK-based Chinese communities are largely invisible in the COVID-19-related public health literature. Limited existing literature on vaccine hesitancy is mostly based on survey research that only provides snapshots of broad trends and patterns and included few samples from the Chinese communities.^6 21 24 25^ A survey-based study conducted in China in March 2020 while the vaccines were still being developed reports high levels of willingness to accept COVID-19 vaccination among the Chinese public due to strong beliefs about the efficacy of COVID-19 vaccines and the perceived large impact of the pandemic.^26^ It also identifies vaccination information based on doctor’s recommendation, vaccine convenience and price as important factors affecting people’s vaccination intention.^26^ The significance of the findings is limited to the specific mainland Chinese context in which strong disease control and public health measures were taken by the authorities. Outside China, few qualitative studies are available to shed light on the complex, multilayered factors and determinants of people’s vaccine hesitancy. Existing literature identifies exposure to vaccine misinformation and the lack of clarity in vaccine-related information as causes of vaccine hesitancy which indicates that communication and information is a key factor shaping people’s attitudes towards and perceptions about vaccination.^14^,^1623^ Therefore this study fills this important research gap and adds value and new knowledge to enquiries into ethnic minority health by focusing on under-researched UK-based Chinese communities. It aims to answer the following research questions:

- What is the understanding of and attitudes towards COVID-19 vaccines within Chinese communities in the UK?
- What informs their understanding of and attitudes towards COVID-19 vaccines and how?

### Study design

The study draws on an interpretive research paradigm which aims at understanding the subjective and socially situated human attitudes and behaviour. In contrast to positivism, a paradigm which seeks to identify the one objective truth, the interpretive paradigm seeks to uncover multiple subjective ‘truths’ that are ‘socially constructed by humans in everyday interaction’.^27^ This study is also informed by community-based participatory research. A Chinese community member working group advised the design, conduct, reporting and dissemination plans of our research. The main empirical part of the study is based on in-depth focus groups with Chinese community members of varied sociodemographic backgrounds which explore their subjective experiences and understanding of COVID-19 vaccination. Focus groups are best suited for generating a rich understanding of participant experiences and beliefs by creating a process of sharing and comparing among participants.^28^ They have also been widely used to explore people’s behaviours and underpinning motivations.^28^ This paper reports on findings from focus group discussions during which we explored participants’ overall understanding of and attitude towards the COVID-19 vaccines, the sources of information about the vaccines and levels of (dis)trust in these sources of information.

### Patient and public involvement

In December 2020, a patient and public involvement (PPI) group from the Chinese community organisation, Wai Yin Society, engaged in discussions with the corresponding author regarding vaccine hesitancy within the community, identifying it as an urgent health concern. In early 2021, the authors and Wai Yin Society submitted an application for a co-design project to investigate the Chinese community’s understanding of COVID-19 vaccines and to promote vaccine uptake, drawing on the PPI group’s experience. On receipt of research funding, a project working group comprising the original PPI group members was set up. The group members were asked to assess the burden and time required to take part in the working group. They participated in three project meetings at the research design stage, data analysis stage and impact delivery stage to advise on the research questions, method (focus group), participant recruitment methods, the design of our COVID-19 vaccine promotional materials (eg, videos of Chinese speaking doctor debunking COVID-19 vaccine misinformation) and our public-facing research dissemination plan (eg, public screening of video and Q&A with Chinese-speaking doctors). The PPI group was not involved in participants recruitment. Members of the working group were given a £60 gift voucher as a token of appreciation.

### Sampling and data analysis

#### Participants recruitment and data collection

In total 154 participants, 119 women and 35 men, took part in our focus groups between March 2021 and November 2021. Their ages ranged between 18 and 87 and their years of residence in the UK ranged from 6 months to 55 years. Participants were educated to different levels from primary school to PhD degree and worked in professional (eg, office worker, teacher, consultant, researcher) and semi-skilled jobs (eg, restaurants, takeaways, shop floor and hair salons). The rest were not in full-time employment as they were retirees, housewives and full-time students. The broad occupational classification was used in data collection to enhance anonymity and encourage participation and disclosure within the close-knit Chinese communities.

Participants were recruited from one northern and one Midland city in England which both have large Chinese communities. Chinese community members were broadly defined as adults who identify themselves as Chinese by ethnicity and who live permanently or temporarily in the UK. In the northern city, participant recruitment was facilitated by Wai Yin Society, one of the largest Chinese community organisations in the UK, from their existing groups and networks (eg, pregnant women’s groups, Tai Chi groups, mental well-being groups). In the Midland city, participants were recruited by two Mandarin and Cantonese-speaking research assistants at local community hubs (eg, Chinese churches, supermarkets, restaurants). The study additionally employed snowballing technique to recruit further participants from our participants’ social networks with the aim of forming natural focus groups. We asked participants from all ages and socioeconomic groups to share our study information with their friends and family who are likely to share the same socioeconomic and demographic backgrounds to ensure broad diversity within the study. Those who were interested were invited to contact the project team or our partner organisation, Wai Yin Society, for further information. Participants were provided with participant information sheets and informed consent forms. The focus groups were then formed on the basis of existing social networks in which participants already knew each other and shared similar socioeconomic and demographic characteristics including age, gender, migration history, occupation and language. For instance, focus group 7 (FG7) comprised mixed-gendered Mandarin-speaking young adults who are friends. While some work as professionals, others are postgraduate students (not in full-time employment). FG13 and FG27 comprised mixed-gendered Cantonese-speaking young and middle-aged adults who were friends and acquaintances. They were professionals who migrated from Hong Kong to the UK within the 2 years prior to the study. Once the minimum participant threshold (4) was reached, the focus group was organised at a mutually convenient time for participants. As the study took place when there was relatively high COVID-19 transmission, all focus groups were conducted remotely via Microsoft Teams, Zoom or WeChat (a Chinese instant messaging and social media app). The choice of the meeting platform was determined by participants’ preferences and accessibility to the platform. Participants were given a £10 gift voucher as a token of appreciation.

Based on our PPI advice, Cantonese, Mandarin or English were offered as language choices for the focus groups. Depending on our participants’ preference, focus groups were conducted in one of these three languages with the exception of group 26 in which a participant preferred to speak in both Mandarin and English. Cantonese was preferred by older participants who migrated from Hong Kong to the UK decades ago and used Cantonese as their main language for communication. Cantonese was also preferred by people recently migrated from Hong Kong to the UK. They were likely to be younger and had shorter residency in the UK. Mandarin was preferred by participants from mainland China and demonstrated strong ties with mainland Chinese media and family and friends. Although many different dialects are spoken in mainland China, because the media system is strictly controlled, the information that mainlanders accessed from the mainstream media is thought to be relatively homogeneous. In this study, the focus groups were moderated by at least one multilingual research assistant who conversed in participants’ preferred language in order to promote open, clear communication and disclosure. Wai Yin staff members involved in participant recruitment also attended the focus group to facilitate introductions and assist in building rapport between the participants and the researcher. In total 29 focus groups were conducted to reach data saturation at which point themes discussed in the focus groups began to be repeated and further data collection became redundant.^29 30^ For participant demographic information, group codes and group composition, please see table 1 below. Further information on aggregated group numbers can be found in online supplemental file 1. Focus group guide can be found in online supplemental file 2. The focus groups lasted on average 65 min and were audio-recorded. The recordings were then transcribed and all personally identifiable information was anonymised.

**Table 1:** Participants’ sociodemographic information

Group code	Age group	Language	Occupational categories	Residency in the UK	GenderMixed (male and female)
FG1	Older adults	Cantonese	Retirees	20 years and over	Mixed
FG2	Older adults	Cantonese	Retirees	20 years and over	Female
FG3	Middle-aged and older adults	Cantonese	Retirees/housewives	20 years and over	Female
FG4	Young and middle-aged adults	Cantonese	Professionals	20 years and over	Mixed
FG5	Middle-aged adults	Mandarin	Semi-skilled	10–19 years	Mixed
FG6	Young adults	Mandarin	Professionals	2–9 years	Female
FG7	Young adults	Mandarin	Postgraduate students/professional	2–9 years	Mixed
FG8	Middle-aged and older adults	Cantonese	Retirees/housewives	20 years and over	Female
FG9	Older adults	Cantonese	Retirees	20 years and over	Mixed
FG10	Young adults	English	Professionals/undergraduate students	20 years and over	Female
FG11	Middle-aged and older adults	Mandarin	Professional/housewives	10–19 years	Mixed
FG12	Middle-aged adults	Cantonese	Professionals	20 years and over	Mixed
FG13	Young and middle-aged adults	Cantonese	Professionals	Under 2 years	Mixed
FG14	Young and middle-aged adults	Mandarin	Professionals/housewife	10–19 years	Mixed
FG15	Middle-aged adults	Cantonese	Professionals	10–19 years	Mixed
FG16	Young adults	Mandarin	Professionals	2–9 years	Mixed
FG17	Young adults	English	Professionals	10–19 years	Mixed
FG18	Young adults	Cantonese	Professionals/postgraduate students	2–9 years	Mixed
FG19	Young and middle-aged adults	Cantonese	Professionals	10–19 years	Mixed
FG20	Middle-aged and older adults	Mandarin	Professionals/housewives	10–19 years	Female
FG21	Young and middle-aged adults	Mandarin	Professionals/housewives	10–19 years	Female
FG22	Middle-aged and older adult	Mandarin	Professionals/retirees/housewives	10–19 years	Female
FG23	Middle-aged adults	Mandarin	housewives/professionals	10–19 years	Female
FG24	Middle-aged adults	Cantonese	Professionals/housewife	2–9 years	Female
FG25	Young adults	English	Professionals/undergraduate students	20 years and over	Mixed
FG26	Middle-aged adults	Mandarin and English	Professionals	10–19 years	Mixed
FG27	Young and middle-aged adults	Cantonese	Professionals	Under 2 years	Mixed
FG28	Middle-aged adults	Mandarin	Professionals	10–19 years	Mixed
FG29	Middle-aged adults	Mandarin	Semi-skilled	10–19 years	Mixed

FGfocus group

#### Data analysis

The focus group data were analysed based on the principles of thematic analysis. The analysis followed the four steps: Familiarising with the data, identifying codes and themes, coding the data and organising codes and themes.^31^ Three authors (QSG, ZG, DW) first read all transcripts to familiarise themselves with the data. The authors read the transcripts several times line by line deductively and inductively identifying a working coding framework constituted by key ideas and recurrent meta-themes in the data. The deductive coding was based on previous literature which identifies broad themes such as sources of information (eg, new media, traditional media, social media, word of mouth). The inductive coding process further developed the themes to subthemes based on the data, for instance, social media was further categorised into WhatsApp, WeChat, Weibo, Little Red Book, Douyin (Chinese version of TikTok) and so on. The coding framework was confirmed in six data analysis meetings involving all other authors (IS, CS, HZ) where new emerging themes were discussed and differences in coding were resolved. QSG, ZG and DW then coded all transcripts in using NVivo software. All authors discussed the selection of the key themes of the paper and quotes from the coded data to be included in thematic sections. A draft paper was sent to two participants who verified our findings before submission. Below we discuss three key interconnected themes from the data.

## Results

### Understanding of COVID-19 vaccines

At the time of our research between March and November 2021, the national COVID-19 vaccination programme which prioritised nine priority groups based on the advice of the Joint Committee of Vaccination and Immunisation was being rolled out across the UK. The rollout started with the most vulnerable and then moved down through age groups and health risk levels.^32^ During this period of time, AstraZeneca’s (AZ) COVID-19 vaccine was found to be linked to very rare cases of blood clotting which can cause fatalities.^33 34^ As this rare condition is seen more often among younger people, the government recommended that healthy people aged 30–39 of age should preferably have a vaccine other than AZ such as Pfizer and Moderna.^33^ On meeting its target of offering the vaccine to all UK adults in July 2021, the government started offering COVID-19 vaccines to 12–15 year olds and booster vaccines to the priority groups in September 2021. In November 2021, booster vaccines, mostly Pfizer and Moderna, were offered to all UK adults due to the wide transmission of the Omicron variant.^32 35^

At the time of our focus groups, some of our participants already had one dose or both doses of a COVID-19 vaccine. Many supported taking the vaccines as ‘the only way out of the pandemic’. Participants generally had a good understanding of the health benefits of the vaccines such as preventing severe diseases and reducing deaths. They also had realistic evaluations of the efficacies of the vaccines as they told us that the vaccines could not completely prevent COVID transmission. However, many unvaccinated, partially vaccinated and even fully vaccinated participants still had concerns about the vaccines. Concerns for vaccine safety, fear of side effects, speed of vaccine development/production were frequently discussed by our participants. They mentioned specific mild side effects including headaches, fatigue, rash, aching arms, allergy, fever and body aches as well as more severe side effects including deaths, blood clots, stroke-like symptoms, infertility and facial paralysis. Many of the actual and perceived mild and severe side effects have been reported in other studies about COVID vaccine hesitancy of the general public.^9 24 36^

In our study, we noted a considerable level of reluctance in some of our participants’ intentions to take the vaccine. Nevertheless, most were cognisant they had ‘no option’ but to take the vaccine to protect themselves and their family, their livelihood or travel internationally to visit family due to the requirement of vaccine passports, a ‘motivational’ factor also reported in another study about cultural and linguistically diverse communities in Australia.^9^

Tell me what I can do if I don’t have the jab? Let’s not talk about national economy, I need to think about my own economy, I must open (the restaurant). It’s not possible to just write off the restaurant business. (FG5_P3) [Focus Group 5 participant 3]

I have no option. As long as it’s not a matter of life or death, going back to China and visiting my family, is my first priority. (FG4_P5)

Others told us that they took the vaccine because their preferred method of protection—wearing facial masks which was perceived to be more effective—was not as widely practised in the UK.

Although I volunteered to get my first jab, I had no confidence in it. I believe wearing a mask and washing hands frequently gives me better protection…In Hong Kong I didn’t need the vaccine because everyone wore a mask. But here people don’t wear masks, and school won’t let children wear masks. I fear this. Children bringing COVID home, and we can’t prevent it. (FG12_P4)

A significant number of participants from various age groups and backgrounds believed that older people and people with underlying health conditions (eg, diabetes, high blood pressure) should not be vaccinated because their bodies are ‘too weak’ to handle the vaccine. This was discussed at length in a third of our focus groups and the belief is congruent with a wider, more holistic traditional Chinese medicine (TCM) concept which prefers using natural remedies and self-nurturing as means to maintain a bodily balance for good health.

According to this cultural belief, all medicines, Chinese traditional or Western, when introduced to the body unnaturally bring side effects as one participant expressed it:

From the point of view of a Chinese family, all medicine has its side effects. You should always avoid having any medication or having any jab. (FG16_P4)

The preference of TCM to Western medicine was found in a Hong Kong-based study which found that although people initially chose Western medicine, they then adopted TCM when they felt that Western medicine was not working.^37^ In the context of COVID-19 prevention and treatment, researchers found that the attitude towards TCM in mainland China was influenced not by rational cognition and logical reasoning but by emotional and cultural factors such as egalitarian, policy endorsement and trust in scientists and healthcare professionals.^38^ A study on a Chinese social media platform also found that the nationalistic framing of China versus the West is significant in the promotion of TCM as a remedy for COVID-19.^39^ Among some of our participants, COVID-19 vaccines which are perceived as unnatural were considered as an external threat to those who are older and weaker. The vaccines were accepted as the lesser of two evils by some who reluctantly received the vaccines. This shows that some cultural and political factors can negatively influence perceptions about COVID-19 vaccines. In over a quarter of our focus groups participants discussed at length the benefits of natural foods that can better nurture the body and strengthen the immune system, offering long-term protection against diseases. Examples mentioned included eating ginger in summer to build up immunity, drinking cooling medicinal herbal tea, taking Chinese medicinal soup to fend off COVID-19 and eating oranges to relieve vaccine side effects.

It’s important to get our bodily balance right. We should have lots of ginger in summer to improve our immune system which can avoid colds and coughs (two known symptoms of COVID). (FG1_P3)

### Sources of information

#### General sources of information

Most participants accessed their COVID-19 vaccine information from a multitude of sources including traditional media and social media, family and friends and medical practitioners (table 2).

**Table 2:** Sources of information reported by our participants

Type of source	Sources of information
News media	BBC, Sky, TV channels from Hong Kong, TV channels from China (CCTV, Guangdong TV station), Community radio
Social media	WeChat public account, WeChat moments, WeChat group, WhatsApp, WhatsApp group, Little Red Book, Douyin, Facebook, Twitter, Instagram, YouTube
Website	Chinese websites, websites searched through Google, Baidu, Sina, Global Times, China News, Apple News, Now News (Hong Kong), KOL online commentators (Hong Kong), Hong Kong TV website, Manchester Evening News
Word of mouth	Family, friend, colleague, medical practitioner, client
Community organisations	Church, community organisation, housing association
Other sources	E-learning papers at hospitals, research articles, workplace emails, NHS app, magazines, TV press conference

Typical comments about these sources are demonstrated below:

Facebook, Google, internet, word of mouth, and a YouTube video of a doctor sharing his opinion and my friend’s mum watched it, bits and pieces and you get all sorts (of information). (FG26_P3)

I’d watch TV. It tells you what vaccine is available. Some of my friends also post WeChat messages about it. (FG2_P4)

Our participants not only accessed information in various types of media (eg, broadcasting, print or digital) but also in different languages (Mandarin, Cantonese and English) originating from different countries and regions including the UK, Australia, France, China, Hong Kong, Malaysia and Taiwan. Older participants (65+), new migrants and people with low English proficiency were more likely to refer to (traditional and new) media sources in Mandarin or Cantonese from their country/regions of origin including WeChat, Weibo, Sina News, Global Times (Mainland China) and Apple News (Hong Kong), typically accessed via personal digital devices. Language barrier is the main reason why Mandarin and Cantonese information was accessed.

I read from 6Park (a Chinese news forum published overseas), YouTube, all in Chinese. My English is poor. (FG5_P5)

I also read news from the internet, links to websites sent by my friends or updates shared by local friends here. I don’t read the BBC because I don’t know English… I read news (about the UK) in Chinese. (FG20_P3)

When participants mentioned the BBC, we specifically asked about BBC’s Chinese service but only two participants said that they used BBC Chinese.

Some also relied on their family members and friends or social media influencers who mediated English information for them:

I mostly hear about the vaccines from my husband. He is a British Born Chinese (BBC) and Hong Kongnese. (FG11_P8)

I heard about it from newspapers…also from my daughter. She works for a pharmacy… (FG8_P5)

I follow a WeChat public account, though I can’t recall the name of the account. The account posted daily updates about COVID in the UK. (FG28_P4)

However, information in Chinese was also accessed by highly educated participants such as postgraduate students and medical researchers. Young British-born participants on the other hand were more likely to access the NHS websites and other English language media such as the BBC, Guardian, Sky, Twitter, Instagram, TikTok, Google, WhatsApp for vaccine information, although some of them were also exposed to information circulated by their family members residing internationally:

I mainly got my information from the NHS website. Or they do like the little NHS information box on like Facebook or Instagram…my Hong Kong family are concerned about what they see on the media…(FG10_P4)

#### COVID-19 vaccine misinformation

The internet and social media have played important roles in providing timely COVID-19 information to the general public and to scientists across the world.^40 41^ However, they can also facilitate the circulation of inaccurate information, misinformation and conspiracy theories.^42 43^ While there is a debate about identifying the ‘intent to harm’ that distinguishes disinformation from misinformation, some argue that the ‘intent’ can be difficult to ascertain.^44^ We use misinformation as an encompassing term in this paper similar to the meaning of the term ‘infodemic’ used by the WHO to describe false or misleading information that can cause harm.^45^ Undoubtedly misinformation has had a detrimental role in vaccine uptake in particular among ethnic minority communities already affected by historic and current discrimination and other structural barriers.^46^ Our data indicate that our participants were exposed to a range of misinformation around COVID-19 vaccines. Participants mentioned 5G, microchip-related government tracking and control, pork gelatine and human foetal cells as vaccine ingredients, but one piece of information—COVID-19 vaccines can cause facial paralysis discussed in a third of our focus groups—seemed to be circulating only within the Chinese communities. Participants traced the origin of the facial paralysis misinformation to social media such as TikTok, Little Red Book (a Chinese short video platform), YouTube and news from Hong Kong and from friends. They also discussed fear and worries which led to vaccine hesitancy:

My relative in Hong Kong refused to get the vaccines. She said it (vaccine) would cause facial paralysis. (FG9_P2)

It might cause facial paralysis. I have a friend who’s had facial paralysis before. He would not get the vaccine. (FG27_P1)

#### Digital access and literacy

Previous studies have identified digital exclusion (eg, limited digital access and low digital literacy) as an important factor contributing to health inequality among migrants during COVID-19.^14 47^ However, in our study, our participants seemed to have wide access to and good use of digital devices such as smartphones, tablets and laptops for textual and audio-visual content, although for older participants the use of social media was mostly limited to making voice and video calls on WeChat and WhatsApp. The latter tend to access COVID-19 information from a narrower range of sources including audio-visual media such as TV and radio news, social media such as WeChat and Douyin (TikTok’s Chinese version), personal contacts and community centres. Some older participants commented that they were not familiar with other text-based media such as websites, mobile apps or social media such as X (formally Twitter), partly because of the language barrier and their age-related visual degeneration. A significant number of younger participants from our study reflected on the vast range of social media information regarding COVID-19 vaccines. Many of them were aware that social media information was not always reliable and some do not use it for ‘serious’ news on health issues.

It’s not true that COVID jabs kill people. I never read this kind of news from Facebook, Instagram or WeChat. All my news about the virus and vaccines is from official news reports. (FG22_P2)

Many younger participants approached social media more strategically, often comparing and contrasting information obtained from social media with information obtained elsewhere. Our participants told us that they crosschecked multiple news websites and evaluated their evidence, sometimes even referring to medical journals.

In fact I don’t like reading English news. I only read on WeChat. I follow a lot of Chinese students who also study in the UK there…then I compare what I read there with what my boyfriend reads from the BBC. They’re pretty much the same. I also check Sina news, and news from the US. (FG7_P8)

I fact-check the origin of the information. I only trust information forwarded from official or government websites. If it’s just some random texts about vaccines being harmful through push notification from WhatsApp or Facebook, normally I wouldn’t believe it. (FG18_P4)

I first heard about it (vaccine) from the BBC. But I was suspicious of their data so I went online to check British Medical Journal…(FG4_P4)

Participants demonstrated good digital literacy as they seemed to be aware that many social media tracked their locations, collected cookies about the content that they browse and pushed similar content to create what is known as an echo chamber, ‘an environment where a person only encounters information or opinions that reflect and reinforce their own’.^48^ However our participants took advantage of some of these functions to actively search for genuine, peer vaccination experiences in their local area shared on social media to help them make their own decisions about vaccination. One participant summed this view up as follows:

The Little Red Book (LRB) tracks our location. If I get invitation today about getting COVID jab, I’d search videos on the LRB and YouTube to check people’s reactions after their jabs, especially those who are in the same age group with me. (FG6_P3)

Three groups in our study consist of participants recently arrived in England under the new Hong Kong British National (Overseas) visa scheme which was opened since January 2021. They told us that they had formed a tight-knit network to share useful peer information about COVID-19 vaccine via WhatsApp. For instance, when the AZ vaccine was first found to be linked with blood clotting risks, they tried to avoid this vaccine. They used their social media network to find out which local general practitioners (GPs) or clinics offered the Pfizer vaccine which was thought to be safer:

When they did Oxford (AZ) vaccine here, we delayed …Eventually we got the jab at our clinic and it was Pfizer! So we immediately messaged our friends on WhatsApp to tell them to sign up there. (FG13_P1)

### Trust and distrust

#### Trust in medical professionals

Existing literature suggests that in the absence of in-depth epidemiological knowledge in the case of COVID-19, trust serves as a sort of heuristic that facilitates action based on the ‘degree to which a trusted individual or organisation recommends it, and as a crucial element of risk communication’.^49 50^ Most of our participants across age groups and country/regions of origin displayed strong trust in medical professionals and scientific experts as credible sources for vaccine information.

I have confidence in the medical care and medicine in the UK. When I received invitation for my jab, I wasn’t concerned at all. (FG3_P5)

They have, you know, been trained in that profession and know more about but medicine and science than Boris Johnson perhaps would. I think I’d feel more comfortable knowing as an actual doctor or professor who was talking about it or informing me about it. (FG10_P3)

On the other hand, our participants questioned the credibility of self-proclaimed medical experts who post/publish information on social media:

I've seen a lot of clips where people playing to be doctors in whatever field disease, viruses whatever, and then provide advice in terms of whether or not we should take vaccine. So I think without a way of validating those people’s profession, I wouldn't believe or trust what they say. (FG26_P5)

#### Trust in government

Our participants from different age groups demonstrated varied levels of trust in the UK government. A significant number of older Cantonese-speaking participants who migrated from Hong Kong decades ago told us that they trusted the UK government as a political authority. There were few references to individual politicians, a point we will return in the discussion, but in general it was the government/authorities some participants’ viewed as trustworthy.

I think the UK government’s vaccine is pretty good. I believe it. If the government asks us to get it, I’ll get it. (FG2_P2)

I believe in the health system in the UK. I’ve been living in the UK for 55 years. I trust it. (FG1_P5)

On the other hand, younger British-born participants, and participants who recently arrived from mainland China were more sceptical of the UK government. This is reflected in their criticism of politicians such as Prime Minister Boris Johnson and the Health Secretary Matt Hancock and their scepticism about the COVID-19 safety measures that the UK government put in place. Primarily accessing mainstream British media, British-born younger participants were exposed to the criticism of politicians by those media. On the other hand, participants who were migrants from mainland China and accessed information mainly from Chinese media, tended to compare the ways in which the UK government and Chinese government handled the COVID-19. Many of them considered the Chinese zero COVID-19 policy more positively. Representative comments from these participants included:

The Health Secretary giving us advice on what to do but going against it [breaking the government’s own rules] yeah so it makes you think whether we should actually trust… (him) (FG10_P4)

I cannot agree with the COVID policy of the government. Easing restrictions is fine but I don’t understand why they don’t ask people to wear masks. (FG26_P1)

My sister said that I shouldn’t take it (the vaccine) because her clinic had a list of conditions, and people with these conditions shouldn’t get the vaccine. The list was provided by the (Chinese) government…Here we were told that people with any conditions can take it…the (UK) government should have given clear guidance about health conditions that are unsuitable for vaccine. (FG20_P2)

The last comment combines the issue of trust in information sources with the question of unmet information needs which is a recurrent theme in many focus group discussions. Some felt that the government should be more transparent about the incidences of vaccine side effects to help people make informed decisions. Others desired trustworthy information in their preferred language provided by the authorities so that any ‘inaccuracies’ in the mediated information that they currently receive from social media influencers, for example, could be avoided.

…if the government, or my GP or the NHS can provide more information (on incidence of vaccine side effects), I believe not only me, but everyone will be more confident in making an informed decision. (FG6_P3)

But sometimes the (social media) influencers that I follow are not specialised in medicine so their information is not always accurate…If there are some authoritative channels that can offer trustworthy news, I think it’ll be great. (FG23_P7)

#### Trust in community organisations, family and friends

When faced with overwhelming and sometimes contradictory and ambiguous information about risks, some of our participants showed strong trust in community organisations and their local Chinese churches in their help-seeking and decision-making:

After I received the vaccine invitation, I rang XX (community organisation) immediately and asked XX (staff), and XX encouraged me to get it. I then rang XX (a friend). She told me there were indeed invitations being sent out and I should get the vaccine if I had been invited. Then I got my jab accompanied by my children. (FG8_P8).

I heard Reverend Luo’s analysis. He said the researchers (of the vaccines) are very senior. Reverend Luo isn’t a doctor. I also heard from my brothers and sisters at the church, and they are doctors. What they said is accurate, so I felt reassured. (FG11_P9)

When dealing with COVID-19 vaccines and their uncertainties about their efficacy and potential side effects, many of our participants resorted to ‘layering up’ multiple sources that they usually trust to ‘firm up’ their decision on whether to take the vaccine. The sources included not only community and faith organisations as demonstrated above but also family, friends, and medical professionals.

My dad’s a doctor, so I guess it’s more trustworthy to listen to him. (FG17_P1)

## Discussion

Our participants generally had a good understanding of the health benefits of the vaccines. However, many still had considerable concern about vaccine safety and effectiveness which may impact on their future vaccine uptake. Our participants accessed COVID-19 vaccine information from a multitude of sources including traditional media and social media, family and friends and medical practitioners.

Young British-born participants tended to access mainstream British media such as the BBC, Guardian, Sky, Twitter, Instagram, TikTok, Google and WhatsApp. Older participants and non-English speaking participants relied more on Chinese media or English language information sources mediated by family and friends and trusted community networks. Their use of media in their preferred language had a direct impact on the information sources that shaped their understanding of COVID-19 vaccines. We also found that many participants with adequate English proficiency still preferred accessing what they perceived to be trustworthy information in their own language. When asked what British media they accessed, most non-British-born participants in our study only identified the BBC, mentioning few others. When participants mentioned the BBC, we specifically asked about BBC’s Chinese service but only two participants said that they used BBC Chinese (https://www.bbc.com/zhongwen/simp). In comparison with other studies on the British public’s attitudes towards COVID-19 and vaccination,^51 52^ there is comparatively little mention or criticism of British politicians in most of our focus groups despite high-profile coverage of controversies over their breaches of social distancing rules by the British mainstream media and social media. We suggest that many in Chinese communities still have limited access to British media generally due to language barriers and media consumption habits. Our British-born, native English-speaking younger participants, however, shared more similarities in their attitudes toward COVID-19 vaccination with the British public because of their regular access to mainstream British traditional and social media.

Most of our participants accessed transnational media and maintained strong ties with overseas family and friends who also shared COVID-19 vaccine information. Obtaining COVID-19 vaccine information from other countries can be problematic as each country had its own guidance.^14^ For example, strong measures such as compulsory facial mask wearing, prolonged lockdowns and mass testing implemented by the Chinese government under its zero COVID-19 policy stood in stark contrast to the measures taken by the UK government. In our study, we traced the origin of the claim of ‘COVID-19 vaccine causing facial paralysis’ to social media from China and Hong Kong. We also found that most of our participants supported the view that very old people and people with underlying health conditions should not be vaccinated, a belief widely held in China and Hong Kong. In contrast to the UK authorities which encouraged vaccination for all, the Chinese and Hong Kong authorities advised against it. It is worth noting that in China only two COVID-19 vaccines—Sinopharm and Sinovac manufactured by Chinese pharmaceutical companies—were available whereas in Hong Kong, Sinopharm, Sinovac and Pfizer were all available. The Chinese central government initially prioritised vaccinating its working-age population (people aged 18–59) who are at high risk and highly likely to spread the virus. On the other hand, some provincial governments did not vaccinate over 60s due to safety concerns because few older people were included in clinical trial.^53^ These rationales may have underpinned different policy recommendations in China. Nonetheless, this has caused confusion and anxieties among some of our participants who needed to navigate complex media sources and negotiate contradictory health advice across national borders, a finding also identified in previous studies about Chinese pregnant migrant women and their maternity care experiences in the UK and the Netherlands.^54 55^ Policymakers and health authorities need to be aware of the influence of transnational media on UK-based ethnic minority communities and address misinformation that is circulating transnationally.^56^

COVID-19 vaccine misinformation can be embedded in cultural beliefs which can lend it plausibility. The misinformation about COVID-19 vaccines being harmful to vulnerable groups was for some reinforced by the TCM concept which prefers natural remedies to ‘unnatural’ vaccines. The vaccines further reinforced some participants’ belief that all medicine has side effects. The misinformation seemingly supported by cultural beliefs may lead to vaccine hesitancy which can further negatively impact on these vulnerable groups who are already at increased risks of contracting COVID-19.^57^ To address misinformation embedded in cultural beliefs, policymakers and health practitioners need to engage dialogically and transparently with communities demonstrating an understanding of their cultural beliefs and practices in order to build trust.^38 58 59^

Many participants raised the issue of unmet information needs as they told us that they desired accurate and transparent COVID-19 vaccine information (eg, incidences of vaccine side effects) through credible/official channels. This corroborates with findings from other studies that government messages about COVID-19 vaccines have not reached various ethnic minority communities because communication was not delivered in a culturally and linguistically appropriate manner.^14 60^ The implication of this for policymakers and health authorities is that they need to build credible channels to better communicate with ethnic minority communities in their preferred languages/dialects. Most of our participants had good access to digital devices especially smartphones, although for older members their independent use of digital devices is limited to voice/video calls and audio-visual content on WhatsApp and WeChat due to lower digital literacy and age-related visual degeneration. Our participants, in particular the younger ones, showed good digital literacy as they strategically used social media to their advantage. This positive finding about digital access and literacy among the Chinese communities indicates some promise for tackling health inequality through digitalisation.^56^ We suggest that a more targeted health communication strategy is required of the authorities. However, our study was unable to recruit extremely marginalised participants such as refugees and undocumented migrants, therefore our findings about good digital media access and literacy should be interpreted with this limitation in mind.

The Chinese communities in the UK are not a homogeneous group, as our study has demonstrated that there are wide diversities in participants’ country/regions of origin, years of residence and main language/dialects spoken which all played a role in shaping their attitudes towards COVID-19 vaccines. For instance, while most of our participants supported the vaccination programme and the underpinning medical expertise, they showed varying degrees of trust of the UK government. Older, Cantonese-speaking participants from Hong Kong who have lived in the UK for a long period of time voiced strong trust in the UK government. However, younger British born and recently migrated participants from mainland China displayed more distrust of the UK government, often citing examples of more successful control of COVID-19 infection in China and Hong Kong and also sometimes identifying particularly untrustworthy politicians. This finding diverges from other studies which have identified distrust of authorities by Black and South Asian communities across the entire community level because of historic and current marginalisation and discrimination.^19 46^ A key learning from Chinese communities is that information translated into preferred languages and communicated via trusted community networks can better reach older members and those who face language barriers.^61^ Policymakers and health authorities should provide funding to make COVID-19 vaccine information available to Chinese community members in their preferred languages, for example, simplified (Mandarin) and traditional (Cantonese) Chinese and disseminate the information through community organisations. Trustworthy, transparent and on-demand information communicated via credible online media and social media is welcomed by younger community members with good digital access and literacy who may act as an information conduit for their older family members. Policymakers and health authorities should consider providing a centralised one-stop online information hub which connects to the online and social media COVID-19 content and widely promoting it among the communities.

### Limitations

Although this study endeavoured to recruit participants from diverse sociodemographic backgrounds, it was unable to recruit marginal participants such as refugees and undocumented migrants who did not want to take part in the study. Our study findings therefore do not represent their experiences and we acknowledge that their understanding or attitude towards COVID-19 vaccination may be determined by very different priorities and concerns such as wishes to remain anonymous from any form of authorities including health institutions. As our study is primarily concerned about participants’ attitude and perceptions about vaccines affected by information and communication factors, we did not collect information on their living or working environment (eg, shared living space with vulnerable people and density). This is an important factor underlying vaccination intent and it requires investigation in further studies. This study only focuses on the experiences of UK-based Chinese communities, therefore our findings have limited generalisability to other UK-based migrant groups who may have lower levels of digital access and greater socioeconomic challenges as factors contributing to health inequality. We encourage future studies to engage with these areas in order to contribute to ethnic minority public health research.

## Conclusion

Our study provides unique insights into the knowledge of and attitudes towards COVID-19 vaccines of UK-based Chinese communities. Our findings demonstrate that members of Chinese communities generally had a good understanding of the health benefits of the vaccines such as preventing severe diseases and reducing deaths. However, many accepted the vaccines reluctantly and still had considerable concern about vaccine safety and efficacy.

Our data reveal that community members had diverse information sources and needs which to a large extent shaped their knowledge of and attitude towards COVID-19 vaccines. Our findings show that although social media can transmit misinformation, their technological advantages such as geotagging and real-time information sharing can enable Chinese community members to enact their agency, actively seeking genuine, peer vaccination experiences in their local area shared on social media to help them access health service.

As transnational media and social networks can significantly shape participants’ perceptions about COVID-19 vaccines, policymakers and health authorities need to address the challenges posed by diverse international information sources including transnational media and family and friends residing overseas. Policymakers and health authorities should dialogically engage with ethnic minority communities through trusted community organisations in order to understand their cultural beliefs and practices, explain possible different policy recommendations and debunk misinformation that is in circulation via the transnational sources. Finally, health authorities need to develop more fine-tuned COVID-19 vaccine communication and promotion strategies, targeting not the entire community but subgroups within the Chinese population in the UK.^62^

## supplementary material

10.1136/bmjph-2023-000658 online supplemental file 1

10.1136/bmjph-2023-000658 online supplemental file 2

## Data Availability

Data are available upon reasonable request.
