# Supplementary material for: Communicating COVID-19 vaccine information to Chinese communities in the UK: a qualitative study of their knowledge, information sources and trust
Source: BMJ Public Health. 2024 Sep 25;2(2):e000658. doi: 10.1136/bmjph-2023-000658 (PMC11816719; doi:10.1136/bmjph-2023-000658)
Supplement: online supplemental file 1 [file bmjph-2-2-s001.pdf]

List of codes

Codes drawn from literature were provided with references. Other codes were generated from the data.

Group number
- 1-29

Type of COVID-19 vaccines
- Pfizer-BioNTech
- Moderna
- Oxford Uni-AstraZeneca
- Sinopharm
- Sinovac
- Janssen
- Medigen
- Sputnik V
- Other

Perceived benefit of COVID-19 vaccines
- Improve immunity and general health
- Prevent infection
- Prevent severe disease
- Reduce death
- Protect family, friends and community
- Improve recovery from COVID-19
- Other

Perceived side effect of COVID-19 vaccines
- Headache
- Shakes (hands)
- Dizziness
- Fatigue
- Drowsiness
- Muscle ache
- Achy arm
- Body ache
- Skin rash
- Fever
- High blood pressure
- Fainting
- Tinnitus
- Allergy
- Chest pain
- Irregular menstrual cycle
- Stomach pain
- Infertility
- Stroke
- Blood clot
- Cough blood

Facial paralysis
Death
Other

Reason of vaccine hesitancy
Side effects [16]
Mis/disinformation [9, 14, 15]
Lack of information or knowledge [9]
distrust of healthcare system
Lack of access to vaccine provider [9]
Other

Concern
Vaccine safety [27]
Vaccine efficacy [4]
Side effects and health problems
Vaccine production processes [4]
Other

Reason for accepting the vaccines
Clinical requirement
Work requirement
Travel requirement [9]
Protect family and friends
Positive vaccine experience of own network [14]
Perceive vaccination as a good collective social cause to resume normality
Other

Vaccine information source
Word of mouth (family and friends) [14, 27]
Community organisations
Medical professionals [27]
Government [16]
NGOs
Pharmaceutical companies
Media (traditional media, new media, social media) [14]
Other

Group description

'Our 29 focus groups encompass a broad range of socio-economic and demographic factors, including age, gender, residency duration, occupation, and language'. Within these 29 groups, 13 groups were Cantonese speaking, 12 groups were Mandarin speaking, 3 groups were English speaking and 1 group was mixed English and Mandarin speaking. In terms of age groups, there were 7 groups of young adults, 8 groups of middle-aged adults, 3 groups of older adults, 5 groups of mixed middle aged and older adults, and 6 groups of young and middle-aged adults. 10 of 29 groups were women-only groups, whereas the rest were mixed groups of men and women. In terms of residency, there were 9 groups of very long-term residents (20 years and over), 13 groups of long-term residents (10-19 years) and 5 groups of medium-term residents (2-9 years), and 2 groups of short-term residents (under 2 years). Regarding occupational classification, 3 groups were retiree-only groups, 11 groups were professionals, 2 groups were semi-skilled workers. The other 13 groups were mixed with some professionals and people not in full-time employment such as housewives, students and retirees. For detailed group information, please see Table 1.
