# Supplementary material for: Communicating COVID-19 vaccine information to Chinese communities in the UK: a qualitative study of their knowledge, information sources and trust
Source: BMJ Public Health. 2024 Sep 25;2(2):e000658. doi: 10.1136/bmjph-2023-000658 (PMC11816719; doi:10.1136/bmjph-2023-000658)
Supplement: online supplemental file 2 [file bmjph-2-2-s002.doc]

**Focus group guide**

1. Have you heard about the COVID-19 vaccine programme? What do you know about it?
2. Which vaccine/s have you heard of? What have you heard about it/them?
3. Where did you hear about the vaccine/s? Do you think what you heard is trustworthy?
4. Do you think the vaccines are un/safe? Why?
5. Are you happy to accept the vaccine when you’re offered it by the NHS? If not, will you consider it at a later stage? When and under what conditions?
6. What will make you more confident about taking the vaccine?

Prompts a) inform you of how to access it? b) give you more information about the approval process and safety issues? c) who do you trust? (NHS, GP, Wai Yin, Family and friends, media)

1. Is there anything you’d like to tell me about the vaccine?
